# Mutual Information for the Detection of Crush

**DOI:** 10.1371/journal.pone.0028747

**Published:** 2011-12-21

**Authors:** Peter Harding, Steve Gwynne, Martyn Amos

**Affiliations:** 1 School of Computing, Mathematics and Digital Technology, Manchester Metropolitan University, Manchester, United Kingdom; 2 Hughes Associates, London, United Kingdom; University of Zaragoza, Spain

## Abstract

Fatal crush conditions occur in crowds with tragic frequency. Event organizers and architects are often criticised for failing to consider the causes and implications of crush, but the reality is that both the prediction and prevention of such conditions offer a significant technical challenge. Full treatment of physical force within crowd simulations is precise but often computationally expensive; the more common method of human interpretation of results is computationally “cheap” but subjective and time-consuming. This paper describes an alternative method for the analysis of crowd behaviour, which uses information theory to measure crowd disorder. We show how this technique may be easily incorporated into an existing simulation framework, and validate it against an historical event. Our results show that this method offers an effective and efficient route towards automatic detection of the onset of crush.

## Introduction

Overloading pedestrian routes can quickly lead to the development of *crush conditions* (should the necessary conditions be evident), as observed in the Hillsborough [1], Station nightclub [2] and Saudi Arabian Hajj [3] incidents. A more sophisticated understanding of how crush conditions form is therefore critical for the architectural design of highly-populated, contained regions (such as ships, nightclubs and stadia), as well as for the planning of events and formulation of incident management procedures. Using this insight, we can begin to understand how and why crush forms as a result of poor design or lack of strategic planning. A first step towards this is a method for *detecting* the early-stage formation of crush, which is the problem we address here.

Computer-based simulation studies are often used to analyse the movement of individuals in various scenarios, often as part of a performance-based design. Such work encompasses the study of historical events [3], the examination of evacuation procedures [4], and the design of aircraft [5]. Existing simulation frameworks include EXODUS [6], PEDFLOW [7] and EVACNET [8], and these offer a range of “real world” features, including exit blockage/obstacles, occupant impatience and route choice [9]. However, the phenomenon of crush is one that has received relatively little attention so far from the designers of evacuation simulations, and many simulations do not explicitly consider the effects of crush.

We therefore seek a method for the detection of crush conditions that may be easily integrated into existing software for crowd simulation. Such a method will have a significant impact on both computer-based evacuation studies and real-time analysis of video images (facilitating, for example, the development of automated crush alarms based on CCTV images). In this paper we give a description of our proposed method, which is based on applying information theory to a system of interacting particles. We show how our method may be easily integrated into an existing simulation framework, and test it using details of an historical event. Simulation results show that our method provides an excellent “early warning” indicator of the emergence of crush conditions.

## Methods

Within an evacuation simulation, the two distinct states of a crowd are characterised by the behaviour of individuals. Under “normal” conditions, crowd flow is highly *ordered*, with the orientation and speed of a specific individual being similar to that of those in their immediate locality. The onset of more *turbulent* flow sees individuals exhibit a marked change in behaviour, as they change speed and alter course in order to avoid others [3]. We therefore wish to identify these distinct states, and achieve this by applying statistical analysis techniques to the movement of individuals within crowds.

In the general case, the Mutual Information (MI) of two discrete time-series variables, A and B, is defined as:
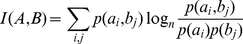
(1)where 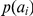, 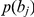, and 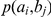 are the individual probability and joint probability distributions of 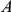 and 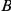. In general terms, MI quantifies the interdependence of two variables; therefore if A and B are entirely independent, then 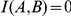, but in all other cases 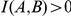. In the context of crowd behaviour, we measure the interdependence of both location and heading over a population of individuals, in order to establish the degree of order within the crowd. An ordered crowd (e.g., one exhibiting stable laminar flow) will have relatively high MI, since individuals are moving in a synchronised fashion. An entirely disordered (i.e. turbulent) crowd will exhibit an MI value of zero, since individuals are acting completely independently of one another. We seek to detect the onset of such turbulence, as an early indicator of crush.

The three variables considered for analysis are the 2-dimensional Cartesian coordinates (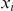 and 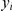) of each individual, 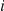, together with their heading, measured in radians (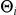). We forego the use of speed within our analysis, as there is often little variation in speed during incidents with high population density. We measure MI using Equation 2, taken from [10]:
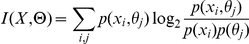


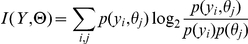


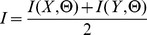
(2)


The base simulation environment used is the Fire Dynamics Simulator (FDS) [11], a fluid dynamics-based model of fire and smoke flow. The FDS+Evac module [12] is an agent-based evacuation simulation extension for FDS, and is based on the established *social forces model* [13], [14] (SFM) of pedestrian movement. An important feature offered by FDS+Evac is that of route selection, which allows the user to embed “knowledge” about available exits into each individual.

Importantly, the evacuation module includes the calculation of *physical forces*, which we will need in order to assess the correlation between crush conditions and mutual information. The contact force suffered by any individual in an FDS+Evac simulation is a summation of the forces exerted upon it by both other individuals and structural components with which it is in contact. The equation which governs the force exerted by individual 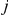 and felt by individual 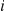 is calculated as:
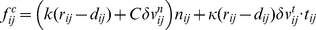
(3)


In this equation, the terms 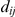 and 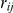 represent the distance between individuals and the combined radii of individuals respectively, so if 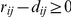 then the individuals are in contact. At this point the radial force constant, 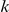, and the frictional force constant, 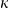, contribute to the force felt by individual 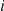. The term 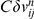 models damping, with 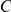 being the damping constant, and is proportional to the difference in the normal velocities of the two individuals, 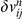. The vector 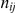 is the unit vector pointing from individual 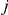 to individual 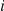. We see then that the first part of the force equation models the direct force resulting from the collision, or persistent contact, between individuals 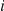 and 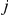. The second part of the equation is similar, but models the *frictional* force produced between individuals, with 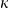 representing the frictional force (or *sliding* force) constant, 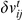 representing the difference in tangential velocities, and 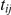 representing the tangential unit vector from individual 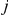 to individual 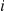. The summation of these two physical forces gives the total physical force exerted by individual 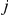 on individual 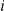. Force exerted by walls or obstacles is calculated in the same manner, with the same constants and variables used in this equation. In what follows, the values of all constants were left at the FDS+Evac default values [15].

We integrate the MI analysis code into the FDS+Evac environment as a set of natively coded (FORTRAN 90) libraries. As the technique is entirely passive, i.e. it will not affect the results of the evacuation, there are no concerns regarding the effect this may have on the outcome of the simulations (although there is clearly a small overhead incurred by the MI calculations). The MI of the system is calculated at every simulation time step, and the results averaged over 100 time steps before being recorded. This equates to one MI reading per second of real-life evacuation time, which gives sufficient granularity. We record the average physical force within a simulation in the same way. In what follows, we use the default FDS+Evac parameter values, as described in [15]. All simulation code is available at http://code.google.com/p/mi-crush/.

### Validation

We first consider the problem of Mutual Information “false positives” (that is, a situation in which “normal” pedestrian flow is incorrectly flagged, via MI measurement, as potentially leading to crush). In order to mitigate against this, we first benchmark the method using a trivial evacuation topology under both emergency and non-emergency conditions. This structure is designed to test the capacity of the MI technique to distinguish between laminar flow and turbulence within the system.

**Figure 1:**
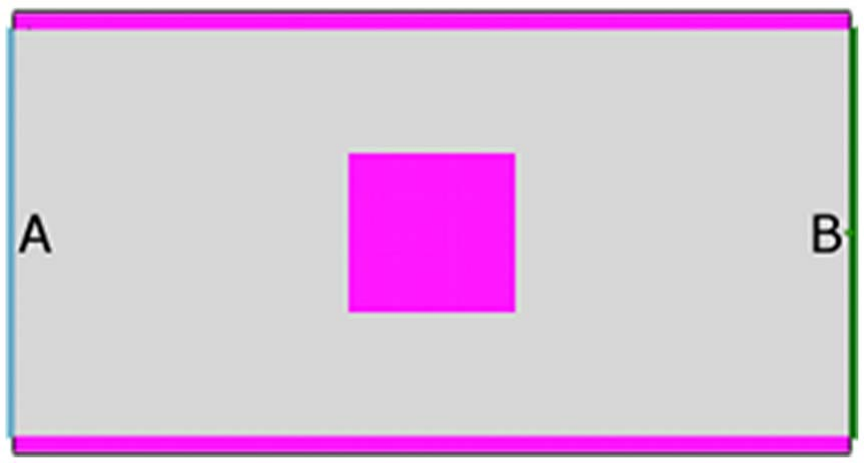
Layout of benchmarking environment. Position A marks the centre of the entry point for pedestrians, and position B marks the centre of the exit.

The topology chosen is a single room, measuring 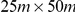, with an exit placed at the east wall, and an identical entrance occupying the same position on the west (Figure 1). The room contains a single, large obstacle, placed in such a way that it disrupts the flow of evacuees. We then perform two sets of runs; the first set tests usage of the structure under “normal” conditions, and the second set tests it during an evacuation situation.

**Figure 2:**
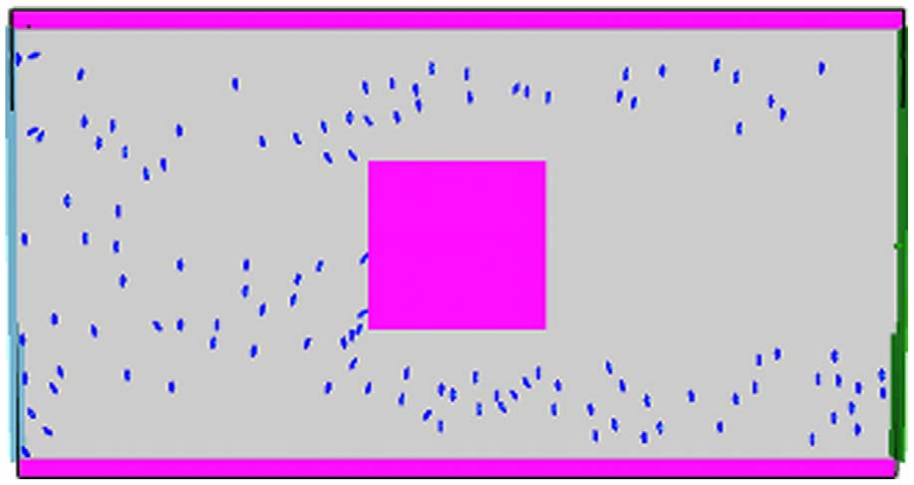
Screenshots of benchmarking simulations. Normal scenario.

**Figure 3:**
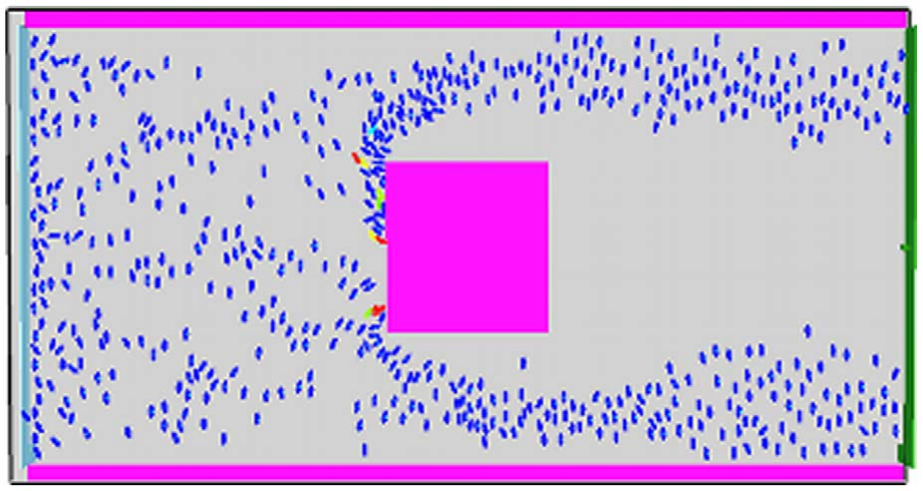
Benchmarking simulations. Evacuation scenario.

For the normal situation, we begin with 20 evacuees at the west of the structure, with additional evacuees added through the west entrance at a rate of 10 evacuees per second of simulation time (Figure 2). The desired leaving speed for evacuees is initially the FDS+Evac default value of 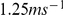. All other parameters are set at the default values. For the simulated evacuation, we aim to overwhelm the capacity of the structure by increasing the input rate to 30 evacuees per second, and increasing the desired escape velocity to 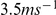 (Figure 3).

**Figure 4:**
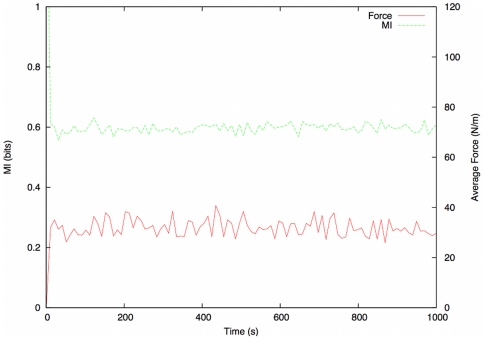
Results of benchmarking simulations. MI (green) and Average Force (red) plotted against time for normal scenario.

We now compare the results of both sets of runs to see if the values for MI differ between them (and thus may be used to identify the different levels of order observed in each situation). Each situation is simulated 50 times, and the results averaged. The MI of the system under normal usage (Figure 4) reaches a stable level of 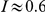 bits after roughly 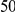 seconds of simulation (after which point there are sufficient individuals in the system to render the results meaningful), and remains at this level for the duration of the simulation. The force figures recorded during this test run are negligible, with the average force reading being 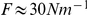 across the population.

**Figure 5:**
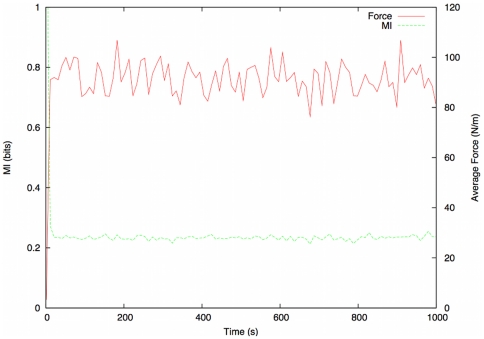
Results of benchmarking simulations. MI (green) and Average Force (red) plotted against time for evacuation scenario.

The results from the simulations in which the structure is overwhelmed (Figure 5) show a far lower basal MI reading, 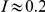 bits, after approximately 50 seconds of simulation time. The force readings, again averaged across all agents, show a significant increase, with an average value of 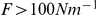 for the majority of the simulation.

These results confirm that MI analysis is relatively insensitive to minor local disorder, but is robust enough to register a lower MI level as disorder in the system increases. We observe a significant difference in MI between normal and evacuation conditions, leading us to conclude that our method is unlikely to generate false positive results, and is capable of detecting the disorder present at the onset of crush.

## Results

In order to test the technique, we choose a well-documented incident that illustrates the significant hazards that an emergency evacuation may present. In 2003, the Station Nightclub (Rhode Island, USA) was the scene of one the worst nightclub fires in recent history, when a pyrotechnic device, used by the rock band Great White, ignited sound insulation foam in the walls and ceiling of the venue. According to the official report into the incident [2], a crush formed at the main escape route within 90 seconds of the start of the fire, trapping patrons inside the club as it filled with smoke. Estimates of the nightclub occupancy vary between 440 and 460; a total of 100 people died during the incident.

We select this particular event on the basis of (a) the existence of a significant amount of professional film footage taken inside the nightclub during the incident - ironically, the film crew was present to record a documentary on nightclub safety, after a fatal incident elsewhere four days previously, (b) availability of supporting witness evidence and other associated documentation, and (c) results from substantial simulation tests using FDS+Evac as part of the subsequent (extensively documented) formal investigation. We therefore have information on the initial distribution of individuals at the beginning of the incident, visual evidence of crush during the incident, and the final locations of each of the victims, as well as an additional set of validated simulations with which to compare our own results. We first ensure that our simulation produces valid outcomes in terms of evacuation profiles (by testing it against the historical event), and then specifically test the MI technique.

### General outcomes

**Figure 6:**
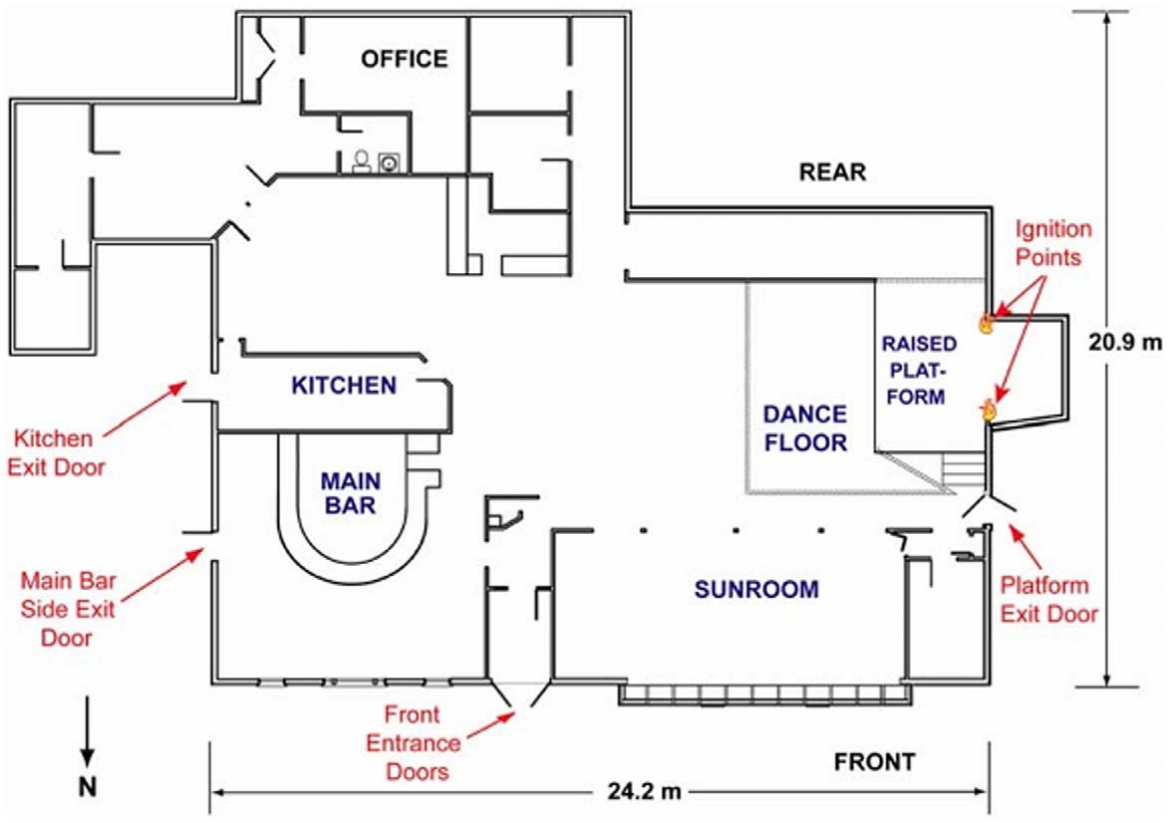
Environment to be simulated. Floorplan of Station nightclub, taken from official report.

**Figure 7:**
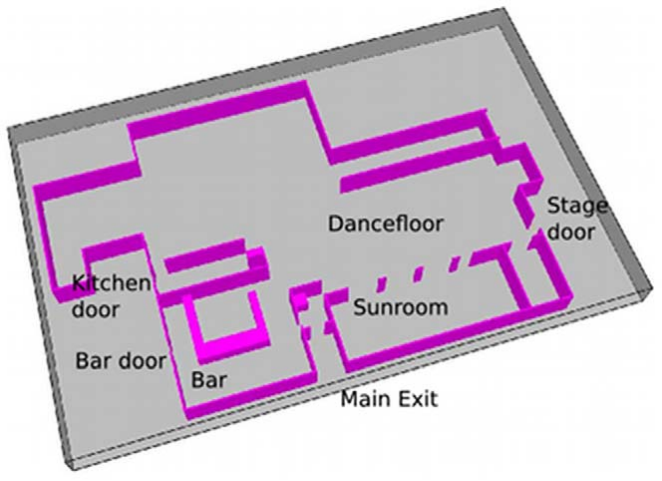
Station nightclub. Rendering in FDS+Evac.

Here, we first ensure that our own simulation produces general evacuation outcomes that are in line with reality (as well as previously validated simulations). We begin by rendering the floor plan of the Station in FDS+Evac, using official architectural plans taken from [2] (Figures 6 and 7). We use a figure of 450 for the number of agents to be simulated, and their initial distribution is specified according to [2] (i.e., with high crowd densities in the Dancefloor and Sunroom areas, and lower densities in other areas).

We run two sets of experiments; the first, *idealised* set is designed to provide baseline evacuation data, and the second set replicates, as closely as possible, the conditions and events in the nightclub during the event. Investigation findings into the spread of the fire suggest that the Stage door became impassable 30 seconds from the start of the incident, so we reflect this fact in our simulation by closing that exit after that period has elapsed. The official investigation was able to identify the exit paths for 248 of the 350 people who escaped from the building. The distribution of evacuees through the three other available exit routes was found to be non-uniform, with estimates of between one-half and two-thirds of patrons attempting to leave via the familiar main exit, rather than the under-utilised (and less familiar) Main Bar and Kitchen doors. Reports suggest that only 12 people left via the Kitchen door during the evacuation. In order to simulate this distribution of path choices, patrons are assigned a probability of knowledge for each exit route. Exactly 12 evacuees are made aware of the existence of the Kitchen exit, and of the remaining patrons, 100% are given knowledge of the main door, 50% are given knowledge of the main bar door, and 25% are given knowledge of the stage door. On the other hand, the idealised evacuation was structured as follows: there was no blocking of the Stage door, and agents in the simulation had full knowledge of all exit routes. This scenario represents the minimum time it would take to evacuate 450 people from the Station Nightclub, with optimum use made of available exit structures and no hindrance from fire, smoke, or unfavourable environmental conditions.

We compare our simulation results with those obtained by the National Institute of Standards and Technology (NIST), and detailed in the official investigation report [2]. In these experiments, NIST investigators used both Simulex [16] and buildingEXODUS [17] to evaluate both idealised and realistic evacuation scenarios. The results obtained were very similar for both packages, so we concentrate on the buildingEXODUS output. Within the “realistic” simulation, occupants were instructed to always select the nearest exit, and the Stage door was also closed after 30 seconds. In the NIST simulation, 91 simulated occupants left via the building front door, which is precisely the number reported in the official investigation. Thirty-five simulated occupants used either the platform door or the kitchen door, which, again, is consistent with the evidence.

**Figure 8:**
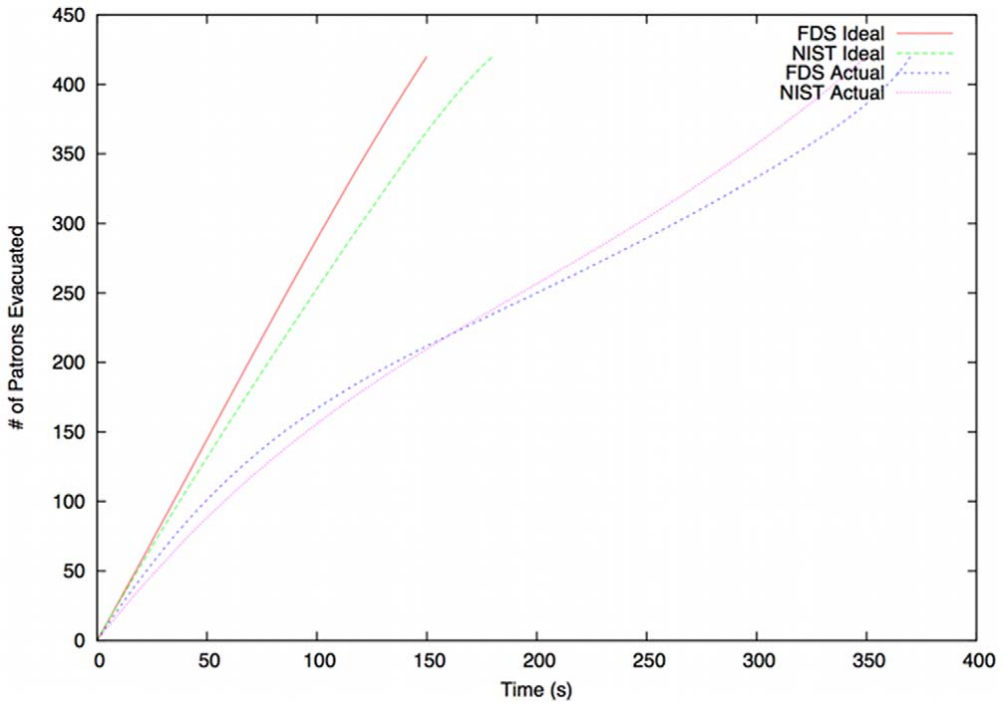
Initial validation results. Comparison of leaving profiles between our simulation (FDS) and official NIST findings.

We therefore conclude that the official NIST simulations provide a sound basis for assessing the quality of our own simulations. The results of the comparison are depicted in Figure 8. We note only that the results obtained (in terms of leaving profiles over time) are very similar to those reported by NIST, which supports the argument in favour of the soundness of our model.

### Crush detection

The next stage is to specifically investigate the emergence of crush in our “real-world” scenario, and to see if crush is easily and reliably detectable using Mutual Information. We repeat the validation experiments described above, but this time we measure the average force (via the built-in FDS+Evac calculation method) and the level of MI within a simulated population of 450 individuals (again, for both idealised (or baseline) and representative evacuation scenarios). For each scenario, the simulation was run 64 times (across a cluster computer), and the results averaged. Recall that in the idealised scenario, all agents have full exit knowledge, and there is no fire blocking the Stage exit, whereas in the representative scenario, exit knowledge is non-uniformly distributed, and the Stage exit becomes blocked by fire after 30 seconds.

**Figure 9:**
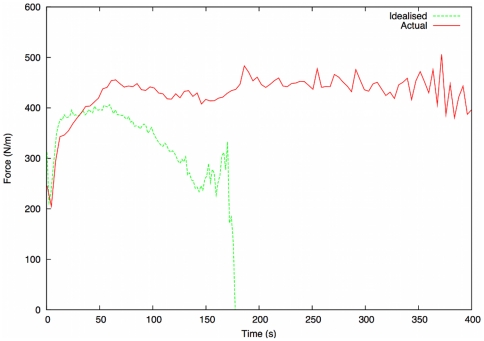
Average force comparison for real and idealised scenarios. Across both scenarios the levels of force initially increase as the evacuation commences, but it rapidly decays during the idealised version of events, since evacuees are more uniformly distributed.

We first consider the results of the force measurements, comparing them with evidence from the investigation. The force measurements for both scenarios are depicted in Figure 9. Across both scenarios the levels of force initially increase as the evacuation commences, but it rapidly decays during the idealised version of events, since evacuees are more uniformly distributed. Force levels drop to zero at around 175 s, when everyone has left the building, which is broadly in line with the findings of the NIST idealised situation simulation (195 s
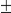
7 s).

In the representative (“real”) scenario (which corresponds to the actual conditions inside the Station), we observe a sharp initial rise in average force, which initially peaks after around 65 seconds. This is directly in line with the findings of the official investigation, which states that a significant crowd crush occurred by the main entrance (where around a third of the fatalities occurred) at the beginning of the time period 71–102 seconds into the fire.

“Prior to 1–1/2 minutes into the fire, a crowd-crush occurred in the front vestibule which almost entirely disrupted the flow through the main exit. Many people became stuck in the prone position in the exterior double doors ([2], p. 232).

The camera angle shifts away from this door after 0:07:33 (0:01:11 fire time) and does not return to the front door until 0:08:04 (0:01:42 fire time). When the camera returns at 0:08:04 (0:01:42 fire time) a pile-up of occupants is visible. Details regarding how the pile-up occurred are not available from the WPRI-TV video; however, the interruption in flow of evacuating occupants apparent [in Figure 6–3] supports the contention that the disruption may have initiated early during the 31 second period when the camera was pointed elsewhere. ” ([2], p. 182)

**Figure 10:**
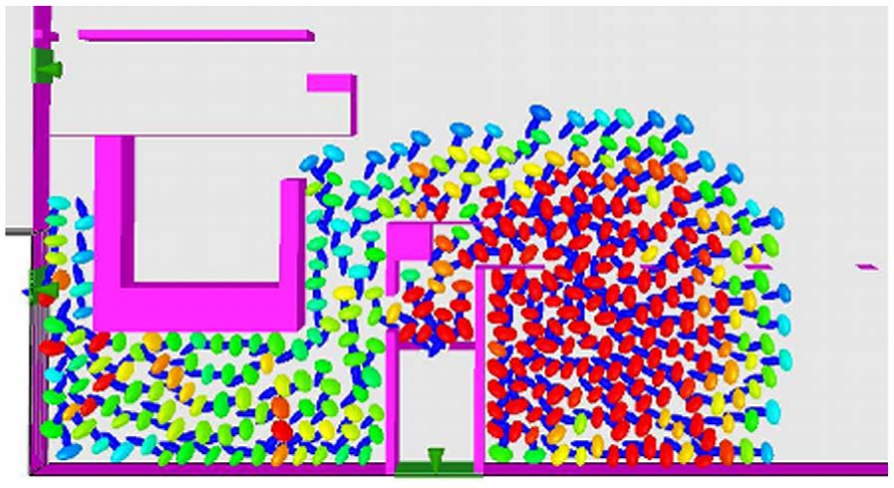
Typical screenshot of our fire scenario simulation after 65 elapsed seconds. This illustrates the significant crush around the main entrance and sunroom area (high levels of force are shown in red).

**Figure 11:**
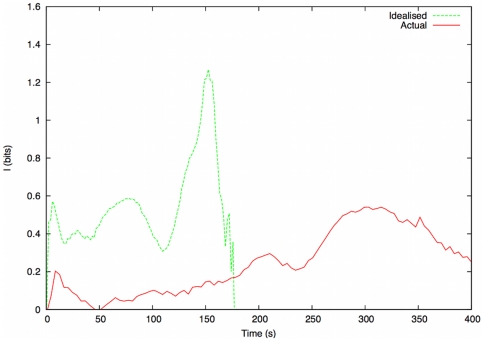
Mutual Information comparison for idealised and representative scenarios. This illustrates the difference between ordered and disordered evacuations in terms of MI.

In Figure 10, we show a screenshot of the simulation after 65 seconds. The MI measurements are depicted in Figure 11. We expect to see, as the simulations begin, an initial rise in the MI of the system. As evacuees prepare to exit the structure they tend towards *alignment*, exhibiting similar escape trajectories to other evacuees in their locale. In a maximally efficient evacuation this period of high order (and high MI) would be sustained throughout, as evacuees would not alter their course in order to increase their chances of effective egress. However, in an evacuation with a great deal of competition, the order in the system quickly breaks down, as the evacuees reposition themselves in order to increase their probability of escape. MI may therefore may be used as an *order parameter*, where falling values of MI signify the breakdown of order within a specific evacuation. We observe marked quantitative differences in the MI readings between the two simulations. During periods of disorder, MI should tend towards zero, whereas, during ordered segments of the evacuation, MI will rise significantly.

In the idealised simulation, we see a sharp initial peak, as individuals all make for the exits at the same time. We then observe a drop, as the evacuees begin to compete for the available exit capacity. An increase in order is seen as one exit route begins to clear, creating the rise in MI at 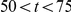, falling back into a state of disorder as the final evacuees clear this (main bar) exit. The MI reading then shows a progressive rise as the final evacuees exit the structure. The sharp drop in MI at the end of the simulation occurs when the number of remaining evacuees falls below some (very low) threshold.

The MI readings obtained from the *representative* simulation of actual events show a far more disordered evacuation, with an initial rise in MI (signifying order) quickly disintegrating into disorder. The MI reading at 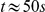 approaches zero; this period of highly disordered evacuation remains as the exits to the structure are overwhelmed (see Figure 10). The exit rate of evacuees during this period is extremely low, which is confirmed by the exit profiles (see Figure 8). The MI level slowly rises towards the end of the evacuation, but, notably, the higher levels of order seen in the idealised evacuation are not reached until 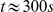, 5 minutes after the start of the evacuation.

**Figure 12:**
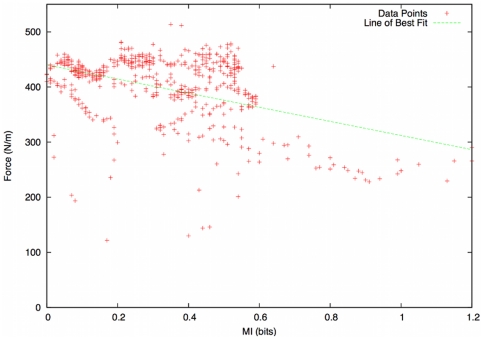
Scatterplot of force versus Mutual Information. This suggests the existence of a statistical association.

We then perform a correlation analysis in order to establish the relationship (if any) between force and Mutual Information. A scatterplot of force versus MI suggests the existence of a statistical association (Figure 12), so we perform a simple linear correlation test. The results of this are as follows:
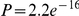


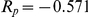



The P-value obtained is much lower than the standard significance level for a two tailed test (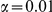), (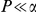), which confirms the significance of the result. The correlation coefficient, 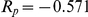, confirms that there exists a negative correlation between MI and force within an evacuation scenario.

## Discussion

Fatal levels of force can emerge within a crowd as a result of pushing, leaning or (less commonly) vertical stacking of bodies. Images of steel barriers bent out of shape (for example, in the aftermath of the Hillsborough disaster [1]) graphically illustrate the extent to which force levels can grow. Fruin reports the results of several studies (either after-the-event forensic tests, or controlled experiments) which suggest that forces exceeding around 1500 N could prove fatal [18]. Crush is therefore an important factor to be considered in simulation studies aimed at improving structural designs or evacuation/control procedures, along with other aspects such as panic or physical obstacles.

Crush detection methods used to date in simulation studies may be classified into two generic groups; *explicit methods* and *implicit methods* [19]. The implicit methodology is the traditional approach, and is still highly popular, being the preferred technique in a large number of simulation models (see [20] for an extensive review). It relies on the expert analysis of factors such as population density and environmental considerations, yielding a human interpretation of the output of the simulation to help determine whether or not crush might have occurred. Although subjective, this method is still popular, because it does not require the use of computationally expensive force calculations, relying instead on human expertise and intuition.

The explicit modelling of crush conditions incorporates an assessment of crush into the model itself, and therefore requires less human analysis than the implicit approach. Usually based on the calculation of Newtonian force values, and operating in 2-dimensional space, explicit methodologies are used to detect the presence of crush conditions in a much more objective fashion. By simulating the physical force exerted by each individual, they calculate the precise amount of force present within a crowd. While the explicit methodologies offer a measure of the forces acting within a crowd, the calculations needed to assess levels of force require much more computer processing power than an implicit method. Experiments show that the computation time required by a model that explicitly quantifies force can be up to 100 times greater than that required by an implicit model [21]. We therefore require a computationally “cheap” alternative if large-scale, iterative studies are to be effective.

In this paper we have described a novel technique for detecting the onset of crush in crowd evacuation scenarios. By calculating the Mutual Information of a system of interacting individuals, we are able to determine the level of order within a crowd. We have shown that consistently low levels of Mutual Information are correlated with high levels of force within a crowd. This method allows planners to quickly and easily incorporate objective measures of crowd disorder and crush into their simulation scenarios. Future work will focus on refinements of the technique, as well as investigation of its “real-world” applicability. A key extension of the method will incorporate partitioning of the simulated space in order to detect the *location* (as well as the existence) of crush. Looking further ahead, we may include the consideration of social and psychological factors within our simulation. We are also interested in the potential for using our technique to analyse real-time video images, with the eventual aim of developing an on-site automatic early warning system for crush and disorder at large-scale events.
